# An AI-Based Digital Scanner for *Varroa destructor* Detection in Beekeeping

**DOI:** 10.3390/insects16010075

**Published:** 2025-01-14

**Authors:** Daniela Scutaru, Simone Bergonzoli, Corrado Costa, Simona Violino, Cecilia Costa, Sergio Albertazzi, Vittorio Capano, Marko M. Kostić, Antonio Scarfone

**Affiliations:** 1 Council for Agricultural Research and Economics, Research Centre for Engineering and Agro-Food Processing, Via della Pascolare 16, 00015 Monterotondo, Italy; daniela.scutaru@crea.gov.it (D.S.); corrado.costa@crea.gov.it (C.C.); simona.violino@crea.gov.it (S.V.); 2 Council for Agricultural Research and Economics, Research Centre for Agriculture and Environment, Via di Corticella, 133, 40128 Bologna, Italy; cecilia.costa@crea.gov.it (C.C.); sergio.albertazzi@crea.gov.it (S.A.); vittorio.capano@crea.gov.it (V.C.); 3 Faculty of Agriculture, University of Novi Sad, Trg. D. Obradovića 8, 21000 Novi Sad, Serbia; markok@polj.uns.ac.rs

**Keywords:** AI, neural network, beekeeping, *Varroa destructor*, scanner

## Abstract

Beekeeping is a crucial agricultural practice that significantly enhances environmental health and food production through effective pollination by honey bees. However, honey bees face numerous threats, including exotic parasites, large-scale transportation, and common agricultural practices that may increase the risk of parasite and pathogen transmission. A major threat is the *Varroa destructor* mite, which feeds on honey bee fat bodies and transmits viruses, leading to significant colony losses. Detecting the parasite and defining the intervention thresholds for effective treatment is a difficult and time-consuming task; different detection methods exist, but they are mainly based on human eye observations, resulting in low accuracy. This study introduces a digital portable scanner coupled with an AI algorithm (BeeVS) used to detect Varroa mites. The device works through image analysis of a sticky sheet previously placed under the beehive for some days, intercepting the Varroa mites that naturally fall. In this study, the scanner was tested for 17 weeks, receiving sheets from 5 beehives every week, and checking the accuracy, reliability, and speed of the method compared to conventional human visual inspection. The results highlighted the high repeatability of the measurements (R^2^ ≥ 0.998) and the high accuracy of the BeeVS device; when at least 10 mites per sheet were present, the device showed a cumulative percentage error below 1%, compared to approximately 20% for human visual observation. Given its repeatability and reliability, the device can be considered a valid tool for beekeepers and scientists, offering the opportunity to monitor many beehives in a short time, unlike visual counting, which is done on a sample basis.

## Simple Summary

A major threat to honey bees is the *Varroa destructor* mite, a parasite that feeds on bee fat bodies and transmits viruses, leading to significant colony losses. Detecting the level of Varroa infestation in the apiary is crucial for defining appropriate intervention strategies and preventing irreparable damage to the colonies. Traditional methods based on manual counting are time-consuming and require meticulous attention. In this study, we tested an AI-based portable scanner for *Varroa destructor* detection. The device operates through image analysis of a sticky sheet placed under the beehive for several days, capturing the Varroa mites that naturally fall. Over 17 weeks, the scanner was tested with sheets from five beehives each week, assessing the accuracy, reliability, and speed of the method compared to conventional human visual inspection. Results show that the system can consistently repeat measurements with high precision, with an error rate in detecting Varroa mites consistently below 1% when there are more than 10 mites per sheet. Given its repeatability and reliability, the device can be considered a valuable tool for beekeepers and scientists, offering the opportunity to monitor many beehives in a short time.

## 1. Introduction

Beekeeping is a multifaceted agricultural activity that offers numerous products and benefits to food production and the environment, primarily through bee pollination. It is estimated that all bee species (about 25.000 among wild and bred) contribute to 70–80% of crop pollination [1]. In this regard, the contribution of honey bees to pollination ranges from 13% in natural ecosystems [2] to 49% in agroecosystems [3]. Pollination promotes vegetable and fruit production, ensures biodiversity conservation, mitigates climate change, and supports environmental health [4,5,6,7]. However, honey bees face several environmental and biological threats, which have become more pronounced in recent years due to climate change acceleration, mass beekeeping, and large-scale transportation of managed bee colonies [8]. These practices, combined with climatic instability, have increased the risk of parasite and pathogen transmission [9,10].

A major threat to beekeeping worldwide, and a significant cause of honey bee colony losses, is the arachnid *Varroa destructor.* This parasite feeds on the fat bodies of larvae and adult bees, reproduces in the brood cells of developing bees, infests them, and transmits viruses [11]. Higher winter temperatures can create more favorable conditions for Varroa reproduction due to the availability of open brood for a longer time. The level of *Varroa destructor* in the apiary should be kept as low as possible to avoid large damages to the colonies; treatments involve chemical applications, including soft chemicals (organic acids like oxalic acid and formic acid) and hard chemicals (synthetic acaricides such as amitraz and fluvalinate) [12,13], as well as biotechnical methods (drone brood removal, worker brood removal, and queen caging) [14,15]. The efficacy of these treatments is highly influenced by climatic condition (temperature and air humidity), presence of brood, and viral loads. The timing of intervention is extremely important because if the number of *Varroa* mites overcomes a critical threshold in the hive, the damage to the colony becomes irreparable [16]. Therefore, identifying the correct moment of intervention in the apiary is crucial for the success of the treatment strategy against *Varroa* [17].

One of the most common methods to determine the critical threshold of intervention is the natural mite fall method [18]. This involves placing a piece of white cardboard coated with a sticky substance at the bottom of the hive. Mites that naturally fall off the bees will land on the board and may be counted [19]. The level of infestation in the colony can be estimated by counting the number of mites on the board fallen over a set period, usually one to three weeks, for colonies in broodright conditions [20]. Generally, a threshold limit is set when the natural fall of mites is ≥10 individuals per day, although this value depends on season, latitude, and beekeeping practices. Other methods for estimating the level of Varroa infestation in a colony include counting the mites present in a sample of adult bees or within a portion of brood. These methods are invasive and almost always involve the death of the bees or brood under investigation. Additionally, detecting the mites in these samples requires time-consuming laboratory techniques and trained personnel, making them rarely applicable in beekeepers’ apiaries. Furthermore, the method based on assessing the number of mites in a sample of adult bees has been found to be not always reliable, depending on the season and infestation level [21]. In contrast, natural mite fall is non-invasive, and its detection, unlike the above methods, can be prolonged and potentially continuous. Mite fall on the bottom boards after treatment can provide useful information on the overall infestation levels of colonies, which may support selection programs or territorial intervention strategies. Currently, counting mites relies on the beekeeper’s visual observation, which can be complicated by the small size of the mites (about 1 mm), the presence of debris, and the high number of mites. This makes visual reading sometimes difficult and prone to overestimations or underestimations [19].

Difficulties in *Varroa* detection highlight the need for technologies that can quickly estimate *Varroa* population [22]. Digital agriculture, particularly AI systems, may provide significant support in this direction. Today, several digital tools are used in beekeeping, and some have been developed to combat diseases such as *Varroa* [23,24,25,26,27,28]. However, although these systems are useful for detecting *Varroa*, they are still at the prototype level, and their use in beekeeping is not documented. Therefore, human visual counting remains the most widely used method. Recently, a scanning device called BeeVS has been developed by the Italian Association for Social Promotion (APISFERO), exploiting artificial intelligence (AI) algorithms for identifying and counting *Varroa destructor* mites. Despite the interesting concept and potential for the beekeeping sector, the performance of the device has not yet been scientifically validated in detail. This work aims to describe the device, assess the repeatability and accuracy of the instrument in counting *Varroa* mites, and compare its performance with human visual counting.

## 2. Materials and Methods

The experiment was conducted between April and August 2024 at the farm of the Research Center for Engineering and Agro-Food Processing of the Council for Agricultural Research and Economics (CREA IT), located in Monterotondo (Rome) 25 m above sea level. The landscape of the farm is characterized by cultivated fields and scattered infrastructures primarily used as offices, laboratories, and storage for agricultural vehicles.

For the study, an apiary was established, consisting of five sensorized Dadant Blatt model beehives, each containing 10 honeycombs fully occupied by honey bees (*Apis mellifera* ligustica Spinola) with queens hatched in 2023. The apiary is part of another scientific activity within the framework of the ProTechBee project, the results of which will be published separately (https://protechbee.crea.gov.it/index.php/it/, accessed on 1 August 2024).

During the trial, a sticky sheet was placed under each beehive to collect *Varroa* mites that fell from the bees. These sheets were replaced weekly over 17 weeks, resulting in a total of 85 sheets used (17 weeks per 5 beehives). Each used sheet was transported to the laboratory, where the BeeVS device was installed for scanning.

### 2.1. BeeVS Description

The BeeVS system consists of a portable scanner trolley that operates on a 12-volt power supply and can be used both in the apiary and in the laboratory. The system is remotely controlled via a smartphone, which functions as a Wi-Fi remote control. Onboard, it features two computers: an Arduino that controls the X and Y movements of a 2 MPX video camera, model ELP USBFHD01M- L60 (Shenzhen Ailipu Technology Co., Ltd., Shenzhen, China) and a Windows 11 mini-PC that manages the acquisition of 60 partially overlapping 50 × 70 mm images. These images are stitched together and sent to the cloud, where they are processed by multiple neural networks. A total of 60 macro photographs are taken, amounting to 120 MPX.

The sticky sheet with debris is inserted into a slot in the trolley, positioned on a black background approximately 100 mm from the camera. Eight LEDs surrounding the camera provide uniformly diffused light to illuminate the sheet. The scanning process takes about 60 s, regardless of the number of *Varroa* mites. Within a few minutes, the *Varroa* count can be accessed on a specific data consultation platform. This platform stores data from all scans, allowing users to consult the statistical trend of *Varroa* for each hive.

### 2.2. Algorithm Description

The Varroa detection algorithm consists of a morphological operator paired with two neural networks that work sequentially. The first is a network with four convolutional layers, and the second is a residual network with 25 convolutional layers. The training was conducted using approximately 65,000 positive samples (*Varroa*) and 1.2 million negative samples (non-*Varroa*), extracted from photos of hundreds of scans performed with multiple BeeVS devices, which were manually annotated. The scans come from various geographical areas and were conducted at different times of the year to ensure good representation of the variability of deposits that can be encountered on the sheet, thus achieving good generalization by the recognizer.

### 2.3. Sampling Methodology

The goal of the study was to evaluate the accuracy of the BeeVS device in counting Varroa mites compared to visual inspection performed by humans. For this reason, on each sampling date, the *Varroa* mites present on the sticky sheet of each beehive were counted through visual inspection and using the BeeVS device. The time required for counting *Varroa* and for scanning the sheet was also recorded.

To assess the reliability of the results obtained using the BeeVS, each sheet was scanned in three different positions: (i) parallel to the scanner plane, (ii) rotated 45° clockwise from the initial position, and (iii) rotated 180° clockwise from the initial position. This procedure was repeated for each sampling date and for each hive to evaluate if the scanning position could affect *Varroa* counting.

Visual inspection was performed by three operators for each sampling date and for each hive to assess the reliability of the results and to determine if visual inspection is influenced by the operator. The accuracy of BeeVS and visual inspection was evaluated by comparing the number of *Varroa* detected using each method to the actual number of *Varroa* present on the sheet (control treatment). This value was determined through a cross-check between the scanner and the visual observation. Each image of the scanner was enlarged onto a 60-inch screen and visually controlled by one trained operator, which added, removed, or confirmed the mites identified. In case of doubt, a direct visual control on the sheet was also performed using a magnifying glass.

### 2.4. Statistical Analysis

Different parameters were analyzed to verify the repeatability, the efficiency, and the accuracy of the BeeVS in comparison with the human visual inspection counting and the real data (control). Statistical analysis was performed utilizing the free software PAST 4.17 released by the University of Oslo. According to the parameter to be analyzed, different statistical approaches were adopted. The following table (Table 1) summarize for each studied parameter the corresponding statistical analysis used:

## 3. Results

The results have been divided into three sub-chapters aimed at describing the repeatability, the efficiency, and the accuracy of the instrument with respect to the visual inspection. This organization facilitates the analysis and interpretation of the data in relation to the study’s objectives.

### 3.1. Repeatability of the Data: BeeVS Efficiency Compared to Visual Inspection

**Figure 1:**
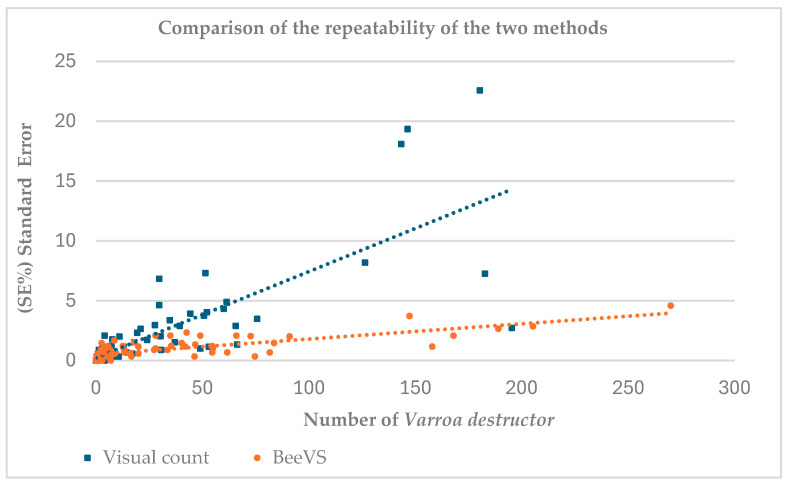
Comparison of visual inspection and BeeVS methods in detecting *Varroa destructor*. R^2^ values refer to the correlation between standard error (SE) and the mean of the *Varroa* counted (three replicates) with the two methods.

Figure 1 shows the comparison between the number of *Varroa destructor* recorded using the visual inspection method (blue squares) and the BeeVS method (orange dots) in relation to the associated standard error (SE) calculated over three replicates for each observation (Figure 1).

The data show a cluster for *Varroa destructor* values below 75, associated with a standard error (SE) under 5%, suggesting good reliability of the replicates in this range for both methods. The dots related to BeeVS (orange) are concentrated in the SE range between 0 and 5 units, indicating low variability in the measurements. In contrast, the squares related to the visual count (blue) show higher SE, with values extending up to over 20 units. This difference is also confirmed by the slope of the regression lines, which is steeper in the visual count. For the BeeVS method, an increase in SE is observed with an increase in the number of *Varroa destructor.*

The number of *Varroa* mites, expressed as the mean of the three replicates, was lower than 200 units with the visual inspection method, while it reached a maximum of 270 with BeeVS. This result highlights the limitation of the operator in detecting a high number of Varroa.

**Figure 2:**
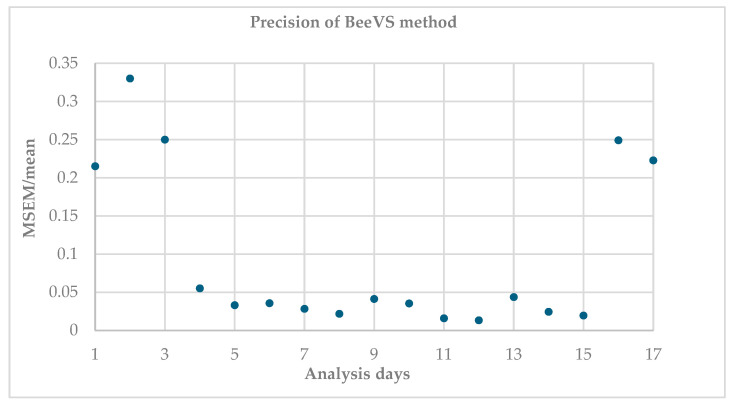
Scatter plot of the precision of the BeeVS method expressed as the normalized ratio among mean standard error and the mean of the replicates (MSEM/mean) for each day of analysis.

Figure 2 shows the ratio between the mean standard error and the mean of the replicates (MSEM/mean), indicating the variability of the measurements between the replicates (acquired with different orientations of the sheet).

Higher values indicate greater dispersion and therefore lower precision. It is observed that this ratio is higher on the first and last days of the analysis, for a total of five dates, corresponding to 29% of the observations. In contrast, 71% of the observations (12 dates) show that the MSEM/mean ratio is almost stable and low, highlighting good replicability of the scanner regardless of the orientation of the sheets.

**Figure 3:**
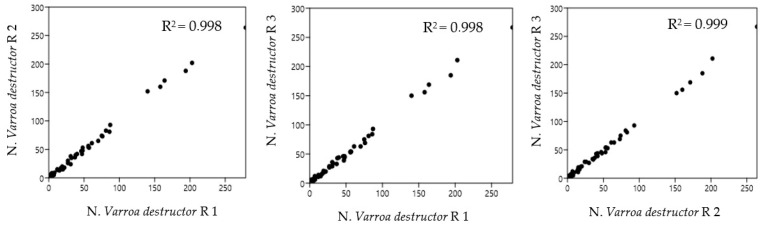
Linear correlation between BeeVS replicates for each observation as a function of sheet orientations (R1 long side of the sheet parallel to the scanner opening, R2 45° rotation from the initial position, and R3 180° rotation from the initial position).

Figure 3 shows the Pearson correlation of the possible combinations between the replicates of the observations, as a function of sheet orientations, acquired with the BeeVS method.

As highlighted by the high value of R^2^, there is a strong correlation among all three combinations of replicates regardless of sheet orientation (R1, R2, and R3), highlighting good repeatability of measurement for the instrument.

### 3.2. Detection Time Efficiency

**Figure 4:**
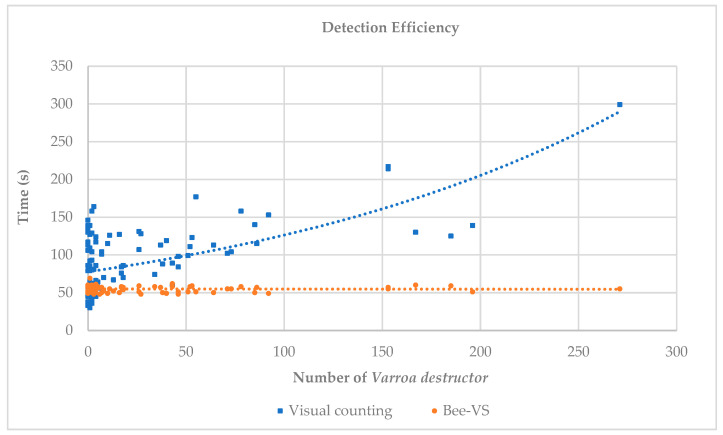
Comparison of the efficiency of the two methods (visual inspection and BeeVS) in the detection time of *Varroa destructor*.

From Figure 4, we can observe the trends of the times related to visual counting and BeeVS counting.

The time required for visual inspection was highly variable (30–160 s) when the number of mites was in the range of 0–10. As the number of *Varroa* increased, the time required for detection increased exponentially. In contrast, the time required for scanning each sheet with BeeVS (orange dots and line) was almost constant and independent of the number of mites present. For all observations, the time used for scanning with the BeeVS was less than 60 s. It should be noted that the times related to the BeeVS scanner represent exclusively the scanning times and do not include the processing times of the algorithm (data availability), which are approximately 10 s.

### 3.3. Accuracy of Varroa destructor Counting Methods, Visual Inspection, and BeeVS Compared to the Real Value

Table 2 shows the accuracy of the two counting methods obtained through the calculation of the standard deviations (Visual vs. BeeVS) with respect to the real data obtained by cross-checking the scan and the adhesive sheet.

The data were grouped into three ranges based on the number of *Varroa destructor* counted. The standard deviation of each observation group was calculated for both methods with respect to the real data. Table 2 shows the results of the statistical analysis using the non-parametric Friedman test for paired samples and the post hoc Wilcoxon test to identify significant differences in the standard deviation of the error.

In the range N ≤ 30, no significant differences were observed between the two methods (visual counting and BeeVS) with respect to the real data. In the range 30 ≤ N ≤ 60, a very significant difference in the standard deviation of the error in the visual count compared to the real data was highlighted (R-Visual = 13.38 **). In the range N ≥ 60, a significant difference in the standard deviation of the error in the visual count compared to the real data was observed (R-Visual = 32.14 *).

By grouping the data measured in five ranges of *Varroa destructor*, the cumulative percentage error in the visual count and with the BeeVS was analyzed (Table 3).

As reported in Table 3, for the 35 sheets that included between 0 and 2 *Varroa* mites, 29 *Varroa* were cumulatively present as detected by the control treatment. In this case, the scanner proved to be much less reliable than the visual count, counting 64 *Varroa* compared to the 21 identified by human observation. The visual inspection underestimated the mites, with an error of 26.45%, while the BeeVS overestimated the *Varroa* with an error of 120.69%.

In the 15 sheets that included between 3 and 10 *Varroa* per sheet, the total *Varroa* present was 76. In this case, the percentage error was similar (26.32% for visual and 22.36% for the scanner). However, in the case of the visual count, it was an underestimation (56), while it was an overestimation in the case of the BeeVS (93). For the other ranges, the percentage error decreased for both methods. The visual inspection error decreased from 18.22% in the range 11 to 50 *Varroa*, reaching a minimum of 14.89% for more than 100 *Varroa*. Regarding the BeeVS method, the error decreased from 0.78% in the range 11 to 50 *Varroa*, reaching a value of 0% for more than 100 *Varroa*.

**Figure 5:**
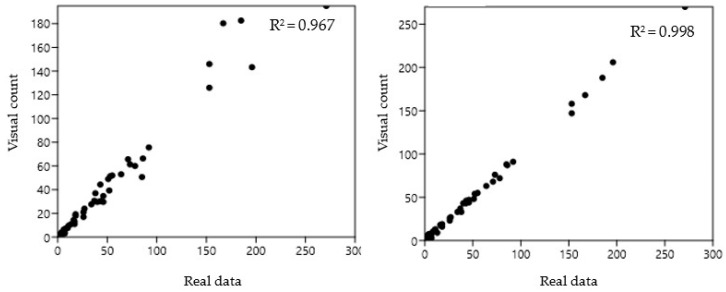
Pearson correlation for *Varroa destructor* counting methods compared to the real data.

An additional comparison was evaluated by correlating the BeeVS and the visual observation with respect to the real data (Figure 5). In this case, the higher reliability of the BeeVS was confirmed by the higher correlation coefficient identified for the BeeVS (R^2^ = 0.998).

**Figure 6:**
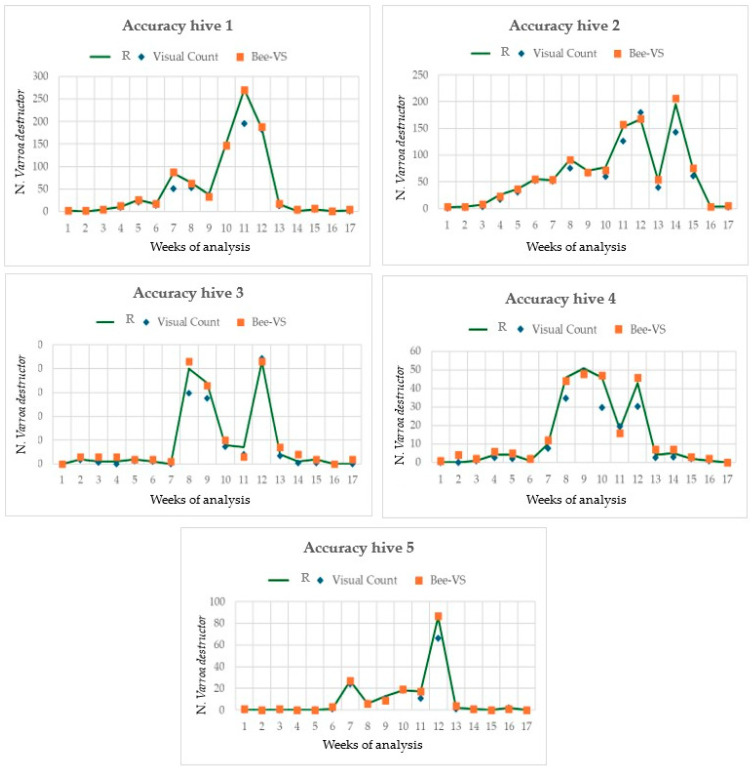
Trend of *Varroa destructor* (real data are Control C—green line) compared with the two methods: visual counting and BeeVS for each hive during the days of analysis.

Figure 6 shows the trends of *Varroa* development along the period of the field test in the five hives monitored and the comparison with the two inspection methods utilized.

The number of mites counted increased from the 7th to the 13th sampling date, although some differences in the trend were observed between the hives. Furthermore, the results highlighted that the accuracy of the visual inspection decreased as the number of *Varroa* increased, while the accuracy of the BeeVS remained quite constant throughout the sampling period. It is important to note that the number of *Varroa* counted varied significantly among the hives. In fact, in hives 1 and 2, the maximum *Varroa* counted was more than 200 mites. In contrast, in hives 3 and 4, it was around 50, and in hive 5, it was 80.

## 4. Discussion

In this study, the efficacy and reliability of an automatic tool for counting the natural fall of *Varroa* mites were determined by comparing the results with human visual counts and real data. Regarding repeatability, the results showed that when the number of *Varroa* per sheet was below 75, the replicates of the two methods (three sheet orientations and three operators) provided similar results, with SE always below 5%. However, when the number of *Varroa* per sheet increased, the human inspection reached an SE four times higher than the BeeVS. These findings highlighted the limitations of visual inspection and the variability of the human eye in accomplishing such a task, as also confirmed by Liu et al., 2023 [23].

Considering the time required for counting the *Varroa* mites, the BeeVS method showed very constant values regardless of the number of parasites present on the sticky sheets. In contrast, the visual inspection highlighted an increase in the time required for counting as the number of *Varroa* increased, following an exponential trend.

Regarding the accuracy of the two methods compared to the real data, the Pearson correlation value was 0.967 and 0.998 for visual and BeeVS counting, respectively. Despite this slight difference, when the accuracy was calculated considering the cumulative number of *Varroa*, the error of the BeeVS method was lower than 1% as the number of mites exceeded 10, reaching a value of 0% for more than 100 *Varroa*. Moreover, a statistically significant difference was found when analyzing the standard deviation of the error in detecting *Varroa* compared to the real data between the two methods, confirming the effectiveness of the BeeVS method compared to visual inspection.

Some authors describe tools developed for a similar purpose showing interesting results. In the study by Sevin et al., 2021 [27], a system called Var-Gor, relying on image analysis, was developed to control *Varroa* mite infestation before and immediately after entry into the hive. It consists of a box located at the hive entrance composed of bee passage tunnels and an autofocus detection camera combined with an interface and supporting image acquisition equipment. The AI technology connected to the system was able to detect existing *Varroa* mites with high accuracy within the trained samples. The authors suggest that the system requires additional training based on the location and color of the mite on the bees, but it may represent a promising smart device for the early detection of *Varroa* mites. On the other hand, Voudiotis et al., 2022 [28] used a camera coupled with a deep learning algorithm to identify bees within brood frames carrying the mite. The camera is placed inside the brood frame and features offline detection in remote areas with limited network coverage or online image data transmission and mite detection on the cloud. The proposed deep learning algorithm uses a deep learning network to detect bee objects and an image processing step to identify the mite on previously detected objects. This system has a total redetection accuracy of bees and *Varroa* of almost 70%. Kryger et al., 2019 [24] developed a similar system based on a portable computer vision system with multispectral illumination and a camera, recording a video sequence of live honeybees *Apis mellifera* for 5–20 min. A computer vision algorithm (Infestation Level Estimator) based on deep learning analysis was used to count the mites to define the infestation level. The system was demonstrated to have good accuracy with minor errors due to mispredictions to be solved with an improvement of the Varroa classifier and the mechanical setup.

Despite the good results obtained by these tools, they remain prototypes that have not yet reached complete reliability and are not commercialized in the beekeeping sector. It is clear that the ultimate purpose of counting the parasites is to estimate and monitor the level of Varroa infestation in a colony. This estimation can have several purposes, including the following: for the beekeeper, to make a decision regarding an anti-*Varroa* intervention; for the breeder, to assess the resistance levels of bee lines; and for the researcher, to evaluate the effectiveness of different types of treatment. The advantages of using the scanner are the accuracy and rapidity of data estimation, especially in cases of high infestation. The tool “learns” continuously, so it is critical to maintain “task forces” such as CREA’s to detect and report to the producer the most frequent errors, currently related to the misclassification of red/purple pollen corbiculae as *Varroa* mites. This means that, in general, the algorithm is well structured at the technical level, including the width of camera focus and type of partition of the scanned area, but it benefits from training in different situations and locations, which may result in different debris compositions.

### General Limitations

Sometimes, the presence of debris on the sheets, originating from bee colony activity (wax corpuscles, pollen, and bee body parts), can influence the correct detection of the mites, as excessive accumulation is likely to invalidate both immobilization and detection. The debris problem can be solved by frequent substitution of the sheets at the bottom, which can vary from a few days to several weeks depending on the season and brood development stage.

On the other hand, physiological factors such as different hygienic behaviors of the colonies and the presence of brood at different stages of development can influence the mite’s fall. Additionally, some mites are lost from the sheets due to the activities of ants or other scavengers. However, these limitations mainly refer to the counting method and the physiology of the honey bees, rather than being caused by the instrument.

## 5. Conclusions

The present work aimed at describing an AI-based scanning device (BeeVS) for *Varroa destructor* detection, assessing the repeatability and the accuracy of the technology in counting *Varroa* mites compared to human visual counting. The BeeVS demonstrated large reliability both in terms of data repeatability and accuracy. Given its repeatability and reliability, the device can be considered a valid tool for beekeepers and scientists, offering the opportunity to monitor many beehives in a short time, unlike visual counting which is done on a sample basis.

## Figures and Tables

**Table 1:** Schematization of the statical analysis utilized according to the parameter evaluated.

Parameter Analyzed—Repeatability	Statistical Analysis Used
Repeatability of the data: BeeVS efficiency compared to visual inspection	Standard error (SE) calculated over three replicates for each observation
Variability of the measurements between the replicates (acquired with different orientation of the sheet)	Normalized ratio among mean standard error and the mean of the replicates (MSEM/mean) for each day of analysis
Correlation between BeeVS replicates for each observation as a function of sheet orientations	Pearson correlation analysis and determination of the correlation coefficient
**Parameter Analyzed—Efficiency**	**Statistical Analysis Used**
Comparison of the time efficiency for the two methods (visual inspection and BeeVS) in the detection of *Varroa destructor*	None, only graphical representation
**Parameter Analyzed—Accuracy**	**Statistical Analysis Used**
Accuracy of the two counting methods (Visual vs. BeeVS) with respect to the real data	Standard deviations, Friedmann test (non-parametric for paired samples) and post hoc Wilcoxson
Accuracy of the two counting methods (Visual vs. BeeVS) with respect to the real data	Cumulative percentage error
Correlation between the two inspection methods and the real data	Pearson correlation analysis and determination of the correlation coefficient
Varroa development along the period of the field test in the five beehives	None, only graphical representation

**Table 2:** Differences in standard deviation of the errors made with the two inspection methods (visual vs. BeeVS) compared to the real data. ** Denotes the presence of significant difference (*p* < 0.01), and * denotes the presence of significant difference (*p* < 0.05) respect the real data.

Range of *Varroa destructor*	N. Obser.	Dev. Standard R-Visual	Dev. Standard R-BeeVS
N ≤ 30	61	2.13	1.75
30 ≤ N ≤ 60	14	13.38 **	2.78
N ≥ 60	10	32.14 *	4.38

**Table 3:** Cumulative percentage error for visual inspection and with the BeeVS method compared to the real data.

*Range of Varroa destructor* Per Sheet	N. Observation	Total Number of *Varroa destructor* Counted with Visual Inspection	Total number of *Varroa destructor* Counted with BeeVS	Real Number (Control)	Error % Visual	Error % BeeVS
0 ≥ N ≥ 2	35	21	64	29	26.45	120.69
3 ≥ N ≥ 10	15	56	93	76	26.32	22.36
11 ≥ N ≥ 50	19	422	512	516	18.22	0.78
51 ≥ N ≥ 100	9	564	684	682	17.26	0.29
N > 100	7	1093	1270	1270	14.89	0.00

## Data Availability

The data presented in this study are available on request from the corresponding author.
